# The role of cervical Electrical Impedance Spectroscopy in the prediction of the course and outcome of induced labour

**DOI:** 10.1186/1471-2393-9-40

**Published:** 2009-09-02

**Authors:** Roobin P Jokhi, Brian H Brown, Dilly OC Anumba

**Affiliations:** 1 Academic Unit of Reproductive and Developmental Medicine, University of Sheffield and Sheffield Teaching Hospitals NHS Trust, Sheffield, UK; 2 Department of Medical Physics and Engineering, University of Sheffield and Sheffield Teaching Hospitals NHS Trust, Sheffield, UK

## Abstract

**Background:**

Previous work by us and others had suggested that cervical electrical impedance spectroscopy (EIS) may be predictive of the outcome of induced labour. We sought to determine which probe configuration of the EIS device is predictive of the outcome of induced labour and compare this to digital assessment by the Bishop score.

**Methods:**

In a prospective cohort of 205 women admitted for induction of labour, we used four probes of diameter 3, 6, 9 and 12 mm connected to an impedance meter to measure cervical resistivity (CR) in Ohm.meters at 14 electrical frequencies and compared their values to digital assessment of the cervix by the Bishop score for the prediction of the outcome of induced labour. We tested the association of labour characteristics and outcomes with CR and Bishop score by stepwise multilinear regression analyses, and the accuracy of prediction of categorical clinical outcomes by analysis of the area under the curves (AUC) of derived Receiver Operator Characteristic (ROC) curves.

**Results:**

Of the four CR probe dimensions studied, only the 12 mm probe was predictive of any labour indices. In the frequency range 19 - 156 kHz, CR obtained with this probe was higher in women who delivered by caesarean section (CS) than those who delivered vaginally, and in labours lasting > 24 hrs. Cervical resistivity at 78.1 kHz best predicted vaginal delivery [optimal cut-off <2.25 Ohm.meter, AUC 0.66 (95% CI 0.59-0.72), sensitivity 71.0%, specificity 56.5%, LR+ 1.63, LR- 0.51, P < 0.01] and labour duration >24 hrs [optimal cut-off 2.27 Ω.m, AUC 0.65 (95% CI 0.58, 0.72), sensitivity 71%, specificity 59%, LR+ 1.72, LR- 0.50, P < 0.05]. In contrast digital assessment by the Bishop score neither predicted vaginal delivery nor the duration of labour. However, Bishop score predicted time to onset of labour > 12 hours and induction-delivery interval < 24 hrs [optimal cut-off ≤ 4, AUC 0.8 (95% CI 0.75, 0.86), sensitivity 77%, specificity 76%, LR+ 3.3, LR- 0.3, P < 0.05] whilst CR did not.

**Conclusion:**

Cervical resistivity appears predictive of labour duration and delivery mode following induced labour. However the low predictive values obtained suggest that its current design proffers no immediate clinical utility.

## Background

Approximately 1 in 5 pregnancies requires induction of labour, a process which carries a high caesarean section rate compared to spontaneous labour [1]. Unwanted outcomes such as caesarean section, prolonged labour, postpartum haemorrhage and traumatic birth often result when labour is induced despite an unfavourable cervix [2]. Pre-labour cervical preparation for birth involves a remodelling process called 'ripening'. Quantifying ripening may enable prediction of the spontaneous initiation of labour, and may inform the timing of induction for clinical indications.

Subjective digital examination of the cervix, often summarized in a composite score such as that described by Bishop [3] is the traditional method of assessing whether the cervix is favourable for induction or not. The clinical parameters that constitute the Bishop Score (BS) include cervical consistency, length, position and dilatation, as well as the station of the fetal presenting part. Although some studies report that the Bishop score correlates with the ease of artificial initiation of labour, the interval to the onset of labour, and the duration of labour [4] several others have found no correlation suggesting limited clinical utility [5,6]. The predictive value of sonographic assessment of cervical length for the onset of labour and labour inducibility is also conflicting [6-10].

Electrical impedance Spectroscopy (EIS) has been employed to study the cervix *in vitro *[11,12] and *in vivo *in non-pregnant [13,14] and pregnant women [15-17]. We reported normal values of cervical resistivity (CR) during pregnancy [15] and have observed that CR values are affected by the diameter of the measuring impedance probe: the fraction of injected electrical current that penetrates cervical stroma increases with increasing diameter [18]. Previous studies have been conflicting regarding whether cervical resistivity is able to discriminate between the ripe and unripe cervix prior to induction of labour [15-17]. These studies were undertaken at varied electrical frequencies and employed measurement probes of different dimensions, making comparisons of studies difficult. We hypothesised that optimising cervical probe dimensions and standardising electrical frequencies at which studies are undertaken may facilitate the identification of any clinical utility for this technology.

Since cervical stromal changes associated with ripening may be paralleled by changes in the cervical epithelium [19] we also hypothesized that smaller measurement probes may capture cervical epithelial resistivity changes with greater sensitivity whilst larger diameter probes would derive cervical stromal resistivity more accurately. We therefore sought to; a) determine cervical tissue resistivity values obtained by four cervical probes with tips of diameter 3 mm, 6 mm, 9 mm and 12 mm; b) assess whether CR obtained with any of the probes correlates with any of the clinical parameters that constitute the Bishop score; c) compare the predictive value of CR to BS for the time to onset of labour, labour duration, ease of labour initiation, successful vaginal delivery, and caesarean section for delayed progress in labour.

## Methods

We recruited 205 women admitted for elective induction of labour at term (> 37 completed weeks gestation). Written informed consent was obtained from each participant. The study was approved by the South Sheffield Research Ethics Committee (Reference number: 06/Q2305/141). Participants were excluded if they had any of the following: abnormal cervical smear in the previous 3 years, previous caesarean section, previous cervical surgery, multiple pregnancy, ruptured fetal membranes, reproductive birth defects, or cervical dilatation > 3 cm. Only women who had had a normal cervical smear in the 3 years prior to the study were recruited.

**Figure 1:**
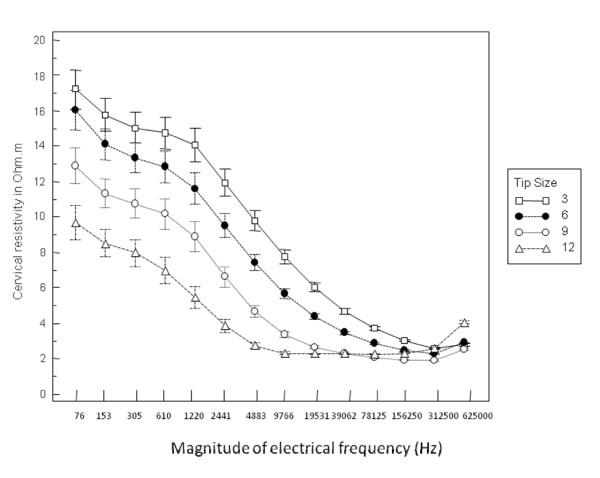
**Mean cervical resistivity for each of the 4 probes**. Mean (SE) cervical resistivity at 14 electrical frequencies obtained with the 4 probes studied for 205 women prior to induction of labour.

At the time of labour induction, a speculum examination was performed and CR was measured as previously described [19]. Two measurements for each of the four probes were taken from each subject. The probe sequence was generated at random. Digital examination of the cervix to assess the Bishop score was performed immediately afterwards [18]. The impedance measuring device consisted of four tetrapolar probes of different sizes connected to a single channel electrical impedance measurement system (Medical Physics and Engineering, University of Sheffield) linked to a computer with a Matlab^® ^software interface (Mathworks Inc., Natick, MA, USA) for data capture and display. The characteristics of the probe tips and electrodes are detailed in Table 1. The basic design of this measurement system *in vivo *has been described previously [14]. Resistivity values were simultaneously obtained at 14 individual electrical frequencies ranging from 76 Hz to 625 kHz (Figure 1) and stored in ASCII format for analysis.

**Table 1:** Characteristics of 4 tetrapolar probes used in the study.

*Name*	*Pitch circle diameter* *(mm)*	*Electrode separation* *(between centres, mm)*	*Electrode diameter* *(mm)*	*Probe diameter* *(mm)*	*Peak current* *(μA)*
3 mm	2	1.41	0.6	5	3.00
6 mm	3	2.12	1.5	9	4.41
9 mm	5.5	3.89	1.5	10	8.33
12 mm	8.5	6.01	1.5	12	12.5

All electrodes were made from 9 ct gold and were housed in a PEEK casing.

The clinical outcomes against which cervical resistivity and Bishop scores were correlated included the following: time to onset of established labour (cervix > 3 cm dilated and at least two uterine contractions every 10 minutes), the duration of labour, the requirement for, and the maximum dose of, syntocinon required for augmentation of labour and successful vaginal delivery. Women whose sole indication for abdominal delivery was suspected fetal compromise were excluded from analysis.

Data were tested for normality of distribution using the Kolmogorov-Smirnov test. Parametric and nonparametric tests as appropriate were used to make comparisons of data obtained with different probes. Correlations between Bishop score and CR values for individual probes on the one hand and the labour characteristics and outcomes on the other were assessed by logistic and stepwise multilinear regression as appropriate. Accuracy of prediction of categorical clinical outcomes by CR and Bishop score was determined from the area under the Receiver Operator Characteristic (ROC) curves, summarized as optimal predictive cut-offs, and positive (LR+) and negative (LR-) likelihood ratios.

## Results

The demographic and clinical details of the study participants are shown in Tables 2 and 3. Figure 1 illustrates the mean (SE) cervical resistivity values obtained with each of the four probes studied for all participants. This demonstrates a fall in the mean resistivity at all frequencies as the diameter of the probe increases.

**Table 2:** Demographic data of participants in study (N = 205)

AGE (MEAN ± SD) *years*	29.0 ± 6.5
BMI (MEAN ± SD) *kg/m*^-*2*^	27.0 ± 6.2
PARITY *n (%)*	
- Nulliparae	94 (45.9)
- Multiparous	111 (54.1)
- Term deliveries only	98 (47.8)
- Pre-term deliveries only	6 (3.0)
- Term + preterm deliveries	7 (3.4)
ETHNICITY *n (%)*	
- Caucasian	190 (92.7)
- South Asian (Indian/Pakistani)	7 (3.4)
- Afro-Caribbean	4 (2.0)
- Oriental	2 (1.0)
- Mixed Race	2 (1.0)
SMOKING *n (%)*	
- No	180 (87.8)
- Yes	25 (12.2)
REASON FOR INDUCTION *n (%)*	
- Post-maturity	69 (33.7)
- Hypertensive disorders	27 (13.2)
- Diabetes Mellitus	22 (10.7)
- Disorders of growth (LGA/SGA*)	19 (9.3)
- Obstetric Cholestasis	8 (3.9)
- Disorders of liquor volume	5 (2.4)
- Reduced fetal movements	3 (1.5)
- Ante-partum haemorrhage	3 (1.5)
- Anti-phospholipid syndrome	3 (1.5)
- Others	46 (22.4)

LGA/SGA: large for gestational age/small for gestational age

**Table 3:** Clinical/outcomes data of participants in study (n = 205)

Gestation at induction in *days*, median (range)	280 (259 - 296)
Bishop score, median (range)	5.(1 -- 10)
Method of induction of labour, *n(%)*	
- Prostaglandin only	41 (20.0)
- Prostaglandin + amniotomy (ARM)	62 (30.2)
- ARM only	102 (49.8)
Syntocinon augmentation, *n(%)*	155 (75.6)
- Dose of Syntocinon, *mu/min, m*ean (SD)	10.6 (6.8)
Duration of labour, *mins, m*ean (SD)	329.2 (194.8)
Labour duration <24 hrs, *n(%)*	
Labour duration ≥24 hrs, *n(%)*	179 (87.3)
	26 (12.7)
Livebirths, *n(%)*	205(100)
Mode of delivery, *n(%)*	
- Spontaneous vaginal delivery	132 (64.4)
- Instrumental vaginal delivery	40 (19.5)
- Caesarean section	33 (16.1)
Indication for caesarean *n(%)*	
- Delayed progress	18 (8.8)
- Unsuccessful induction of labour	2 (1.0)
- Suspected fetal compromise	13 (6.3)

There was no correlation between CR obtained with any of the four probes and the Bishop score. Unlike the other three probe diameters, lower resistivity values were obtained with the 12 mm probe tip in the range of 9.8 - 78.1 kHz in women who were parous compared to nulliparous women (mean CR at 19.5 kHz 2.15 vs. 2.38 Ω.m, P < 0.01).

### Prediction of induced labour by CR vs. Bishop scores

Cervical resistivity obtained with the 3, 6 and 9 mm probes did not correlate with any labour characteristics whilst data obtained with the 12 mm probe did. The latter are compared to the Bishop score below for prediction of time to onset of labour, labour duration, induction-delivery interval and successful vaginal delivery.

### Prediction of time to onset of labour (labour latent)

The pre-induction Bishop score showed a large independent correlation with the time to onset of labour whilst CR did not at any of the frequencies studied (Table 4). The best accuracy for predicting labour latent greater than 12 hours was achieved by the Bishop score at an optimal cut-off value of ≤ 5 (Table 4).

**Table 4:** Prediction of time to onset of labour > 12 hours by the Bishop Score vs. cervical resistivity (CR) measured with a 12 mm probe (data shown for 4 frequencies 9.8-78.1 kHz).

	*Correlation coefficient*	*AUC (95% CI)*	*Optimal cut-off value*	*Sensitivity (95% CI)*	*Specificity (95% CI)*	*+LR*	*-LR*	*PPV(%)*	*NPV (%)*
**Bishop score**	-0.620**	0.85 (0.80-0.90)**	≤ 5	87.9 (77.5 - 94.6)	67.7 (58.8 - 75.9)	2.72	0.18	59.2	91.3
**Frequency of CR**									
**9.8 kHz**	0.044	0.53 (0.45-0.60)	>1.69 Ω.m	95.2 (86.7 - 99.0)	18.03 (11.7 - 26.0)	1.16	0.26	37.5	88.0
**19.5 kHz**	0.029	0.52 (0.45-0.59)	>2.29 Ω.m	46.03 (33.4 - 59.1)	64.75 (55.6 - 73.2)	1.31	0.83	40.0	70.0
**39.1 kHz**	0.033	0.48(0.40-0.55)	>2.15 Ω.m	49.21 (36.4 - 62.1)	61.5 (52.2 - 70.1)	1.28	0.83	39.7	70.1
**78.1 kHz**	0.013	0.51(0.44-0.59)	>2.53 Ω.m	26.98 (16.6 - 39.7)	81.97 (74.0 - 88.3)	1.50	0.89	43.6	68.5

CR cervical resistivity, AUC Area Under the ROC Curve, LR Likelihood ratio, PPV positive predictive value, NPV negative predictive value, ** P < 0.01

### Prediction of duration of labour

**Figure 2:**
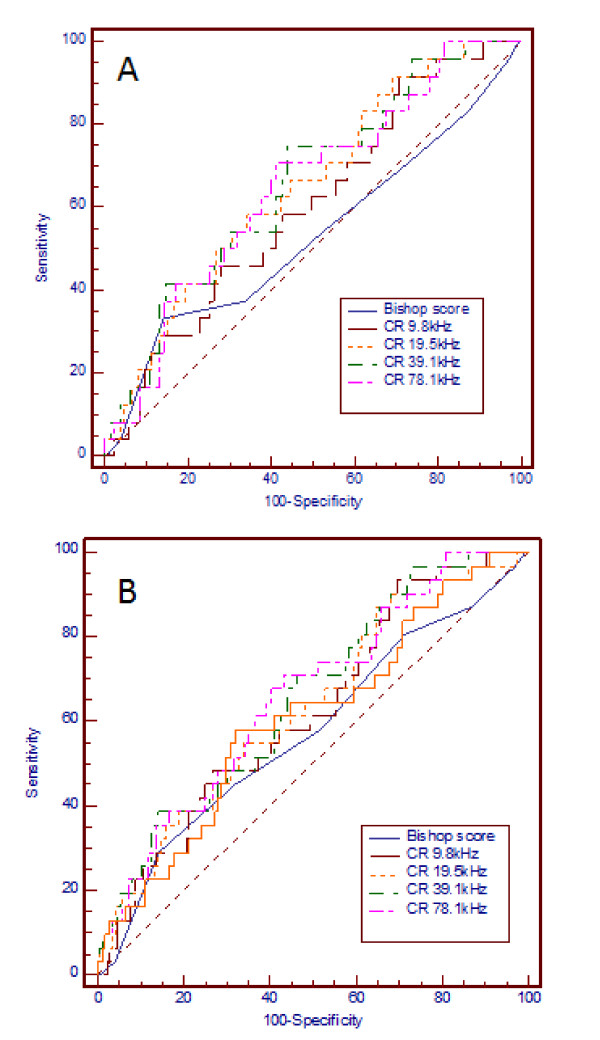
**Prediction of labour duration and vaginal delivery**. **a **Prediction of labour duration >24 hrs by Bishop score vs cervical resistivity with 12 mm probe. ROC curve showing prediction of duration of labour >24 hrs by Bishop score assessment vs cervical resistivity obtained with a 12 mm probe (the AUC was significantly > than the area under the nondiagnostic line). **b**: Prediction of vaginal delivery by Bishop score vs cervical resistivity with 12 mm probe. ROC curve showing prediction of vaginal delivery by Bishop score assessment vs cervical resistivity obtained with a 12 mm probe. Only the AUC for CR 9.8 to 78.1 kHz was predictive of vaginal delivery (significantly greater than the nondiagnostic line, P < 0.01).

Cervical resistivity, at each of the 4 frequencies between 9.8 and 78.1 kHz showed a small correlation with the duration of labour in women who achieved vaginal delivery whilst the Bishop score did not (Table 5). Analysis by stepwise multiple regression demonstrated independent correlations of duration of labour with parity (coefficient of determination R^2 ^= 0.0489, R^2^-adjusted = 0.039, P < 0.05) and with CR (P < 0.05). Accuracy of prediction of labour duration >24 hrs by CR is shown in Table 5, Figure 2a). Unlike CR the Bishop score was not predictive of labour lasting >24 hrs.

**Table 5:** Prediction of labour duration ≥ 24 hrs -- Bishop score vs. cervical resistivity with 12 mm probe (data shown for 4 frequencies 9.8-78.1 kHz).

	*Correlation coefficient*	*AUC (95% CI)*	*Optimal cut-off value*	*Sensitivity % (95% CI)*	*Specificity % (95% CI)*	*+LR*	*-LR*	*PPV (%)*	*NPV (%)*
**Bishop score**	-0.05	0.54 (0.47 - 0.61)	≤ 3	34.6 (17 - 56)	86.0 (80 - 91)	2.46	0.76	27	90
**Frequency of CR**									
**9.8 kHz**	0.09	0.60 (0.53-0.67)	>1.93 Ω.m	92 (70.8 - 98.6)	29 (24.6 - 38.9)	1.33	0.29	15.3	96.2
**19.5 kHz**	0.14*	0.65 (0.58-0.72)*	>2.30 Ω.m	58.3 (37.0 -- 78.0)	66 (58.0 -- 73.0)	1.70	0.63	19.0	92.0
**39.1 kHz**	0.16*	0.66 (0.59 --0.73)*	>2.25 Ω.m	75.00 (49.8 - 89.2)	56.00 (48.3 - 63.5)	1.70	0.45	19.0	94.2
**78.1 kHz**	0.16*	0.65 (0.58-0.72)*	>2.27 Ω.m	70.8 (45.1 - 86.1)	58.9(52.3 - 67.3)	1.72	0.50	19.1	93.6

CR cervical resistivity, AUC Area Under the ROC Curve, LR Likelihood ratio, PPV positive predictive value, NPV negative predictive value, * P < 0.05.

### Prediction of vaginal delivery

Cervical resistivity, between 9.8 and 78.1 kHz, was predictive of vaginal delivery whilst the Bishop score was not (Table 6). The best accuracy of prediction of vaginal delivery was achieved at 78.1 kHz (Table 6, Figure 2b) with LR+ of 1.63, at an optimal cut-off tissue resistivity value of < 2.25 Ω.m. At this frequency stepwise logistic regression analysis (covariates included in model: parity, Bishop score and CR) demonstrated significant independent prediction of vaginal delivery (OR 3.9, 95% CI 1.6 to 10.0, P < 0.01).

**Table 6:** Prediction of vaginal delivery -- Bishop score vs. cervical resistivity with 12 mm probe (data shown for 4 frequencies 9.8-78.1 kHz).

	*AUC (95% CI)*	*Optimal cut-off value*	*Sensitivity % (95% CI)*	*Specificity % (95% CI)*	*+LR*	*-LR*	*PPV (%)*	*NPV(%)*
**Bishop score**	0.57 (0.50- 0.64)	≤ 3	30.30 (15.6 - 48.7)	86.0 (79.8 - 90.8)	2.16	0.81	29.4	86.5
**Frequency of CR**								
**9.8 kHz**	0.62 (0.55-0.69)*	<1.92 Ω.m.	93.55 (78.5 - 99.0)	30.36 (23.5 - 37.9)	1.34	0.21	19.9	96.2
**19.5 kHz**	0.63 (0.56-0.70)*	<2.03 Ω.m.	87.10 (70.1 - 96.3)	35.12 (27.9 - 42.8)	1.34	0.37	19.9	93.7
**39.1 kHz**	0.66 (0.58-0.72)**	<2.24 Ω.m.	70.97 (52.0 - 85.7)	53.57 (45.7 - 61.3)	1.53	0.54	22.0	90.9
**78.1 kHz**	0.66 (0.59-0.72)**	<2.25 Ω.m.	70.97 (52.0 - 85.7)	56.55 (48.7 - 64.2)	1.63	0.51	23.2	91.3

CR cervical resistivity, AUC Area Under the ROC Curve, LR Likelihood ratio, PPV positive predictive value, NPV negative predictive value, ** P < 0.01 level, * P < 0.05.

### Prediction of delivery within 24 hrs of assessment

**Figure 3:**
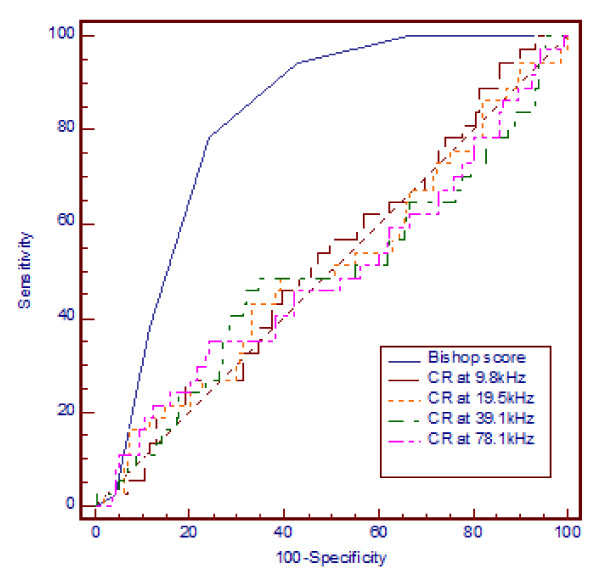
**Prediction of induction-vaginal delivery interval < 24 hrs Bishop score vs cervical resistivity with 12 mm probe**. ROC curve showing prediction of induction-vaginal delivery interval < 24 hrs by Bishop score assessment vs cervical resistivity obtained with a 12 mm probe at 9.8 to 78.1 kHz. Only the AUC for Bishop score was predictive of delivery within 24 hrs of assessment (significantly greater than the nondiagnostic line, P < 0.01).

Overall, the Bishop score was highly predicted of induction to delivery interval of less than 24 hrs [optimal cut-off = 4, AUC 0.8 (95% CI 0.75, 0.86), sensitivity 77%, specificity 76%, LR+ 3.3, LR- 0.3, P < 0.05] whilst CR was not at any study frequency (Figure 3).

### Syntocinon use for augmentation of labour

Cervical resistivity at 78.1 kHz with the 12 mm probe was significantly higher in women who required syntocinon augmentation of labour (n = 147) compared to those who did not (n = 58) (mean ± SE: 2.30 ± 0.04 vs. 2.18 ± 0.05 Ω.m, P < 0.05, respectively). In contrast the Bishop score did not differ (5.42 ± 0.15 vs. 5.20 ± .0.24, P = 0.44 respectively). Neither the Bishop score nor CR predicted the need for syntocinon acceleration during induced labour.

## Discussion

This is the first study comparing digital assessment of cervical ripening to cervical electrical resistivity measured by a bio-impedance technique for the prediction of the outcome of induced labour. We have demonstrated that CR obtained with largest probe size studied - the 12 mm diameter probe - correlated with parity, the duration of labour and successful vaginal delivery in the frequency range 9.8-78.1 kHz. This correlation suggests that impedance spectroscopy of the cervix reflects electrical properties of the tissue which may be relevant to the physiology of normal and dysfunctional labour. However the predictive likelihood ratios attained were low, precluding its immediate application in clinical settings to predict the outcome of induced labour. The Bishop score prior to induction did not correlate with the duration of labour and was not predictive of vaginal delivery. However it was highly predictive of time to onset of labour greater than 12 hours and of delivery within 24 hours of the assessment.

Consistent with our original hypothesis based on computational modelling [12,18] only data obtained with the widest diameter probe (12 mm) correlated with parity and duration of labour, and modestly predicted labour duration > 24 hours and vaginal delivery. The greater inter-electrode distance on the large diameter probe ensured that a higher fraction of injected electrical current penetrated cervical stroma to capture its properties via the sensing electrodes, the stroma of the cervix being the principal site of the remodelling process prior to birth [19-24]. Cervical resistivity may better assess cervical stromal "compliance" than digital assessment, and high cervical stromal resistivity may reflect higher resistance to stretch, which may contribute to labour dystocia, fetal distress and caesarean delivery. Consistent with this thesis, previous studies have shown a positive correlation between cervical collagen concentration and the duration of labour, [24,25]. A limitation of our study was that the numbers of women who required caesarean section for failure to progress and failed induction were too small for us to determine any correlation between CR and caesarean delivery solely for these indications.

We observed a progressive fall in mean cervical resistivity at all frequencies as probe dimensions increased, consistent with our previous observations [18]. The smaller probes are more sensitive to superficial epithelial tissue changes and demonstrate higher resistivity values at low frequencies but do not interrogate deeper sub-epithelial tissues well. The failure of the 3, 6, and 9 mm diameter probes to correlate with labour outcomes whilst the largest probe did, suggests that stromal rather than epithelial tissue elements influence the remodelling processes associated with birth.

The poor predictive value of the Bishop score for successful vaginal delivery in our series agrees with several other reports [5,6,26] but conflicts with others [10,27]. All studies demonstrate that Bishop scores yield low positive predictive likelihood ratios for abdominal delivery, highlighting the limited utility of this assessment. We have observed that the Bishop score is highly predictive of time to onset of established uterine contractions and, consequently, of the induction-delivery interval whilst CR is not. Additionally we have found no correlation between CR values and the Bishop score in contrast to a previous study at a single frequency that suggested an inverse correlation [16]. Taken together, our observations suggest that the Bishop score reflects cervical readiness for labour while CR reflects potential cervical compliance during labour.

The limited predictive value of CR for vaginal delivery suggests that substantial device enhancements would be required before this technique may find clinical utility. Factors which may increase the variability of current impedance measurement probes include the degree of probe pressure on the cervix [28] and the presence of cervical mucus [29]. In one study, increasing the pressure of the probe on cervical tissue increased tissue resistivity by up to 80%, potentially higher than any pathological or physiological changes of interest [28]. With regard to our current studies, if the CR changes associated with pre-labour remodelling are of a lesser magnitude than may be caused by probe pressure on the cervix, any correlation between CR and labour duration or outcomes may be lost or attenuated. The effect of conductive fluid such as surface mucus on trans-epithelial impedance measured using a tetrapolar probe may be counteracted by the use of a guard electrode [29]. Epithelial tissue boundaries such as the squamo-columnar junction of the cervix modify and confound the impedivity spectra obtained across them [29]. Larger probes are more likely to measure across the squamo-columnar junction given the limited surface area of the ectocervix. We may therefore have underestimated the performance of the 12 mm diameter probe.

## Conclusion

Cervical EIS appears a potentially useful diagnostic tool for assessing pre-labour cervical remodelling and for predicting some of the outcomes of induced labour. Although we demonstrated small correlations between cervical resistivity and duration of labour and mode of delivery for the large 12 mm diameter measurement probe, the device performance did not achieve sufficient accuracy to enable employing this tool for clinical purposes at present. Further improvements in the design and application of this technique may enhance its potential application to clinical use.

## Competing interests

The authors declare that they have no competing interests.

## Authors' contributions

All authors have contributed to and approved the final manuscript. DOCA and BHB conceived and designed the study. RPJ and DOCA developed the methodology and RPJ acquired the data. DOCA and BHB conducted the statistical analysis. RPJ and DOCA wrote the first draft of the paper. All authors contributed to writing the manuscript and have approved the final version.

## Pre-publication history

The pre-publication history for this paper can be accessed here:

http://www.biomedcentral.com/1471-2393/9/40/prepub
